# Health Related Quality of Life, an appropriate indicator to assess the impact of morbidity management and disability prevention activities towards elimination of lymphatic filariasis

**DOI:** 10.1186/1475-2883-6-8

**Published:** 2007-08-28

**Authors:** A Krishna Kumari, K Krishnamoorthy, KT Harichandrakumar, LK Das

**Affiliations:** 1 Division of Health Economics and Disease Burden, Vector Control Research Centre, Pondicherry, India; 2 Division of Clinical Epidemiology and Chemotherapy, Vector Control Research Centre, Pondicherry, India

## Abstract

**Background:**

Lymphatic filariasis has been identified as one of the six diseases that can be potentially eliminated. Global programme to eliminate lymphatic filariasis has been launched, applying principal strategies of mass drug administration to interrupt transmission and morbidity management to prevent disability. The strategy for mass drug administration has been clearly laid out and guidelines have been well documented for implementation, monitoring and evaluation of the programme but such a guideline is warranted for morbidity management and disability prevention activities.

**Discussion:**

Health Related Quality of Life, a multidimensional construct referring to patients' perceptions of the impact of disease and treatment on their physical, psychological and social function and well being is crucial in the evaluation of health care interventions. Lymphatic filariasis has a wide clinical spectrum and disability is more pronounced in the advanced stages of lymphoedema and hydrocele. Since the advanced stages of lymphoedema are not reversible, morbidity management and disability prevention activities can lessen the disabilities due to secondary infections and there by improve the quality of life of the patient. Thus, an improvement in quality of life is considered to be important as a primary outcome in the determination of therapeutic benefit. Therefore it can be used as an indicator to assess the impact of morbidity management and disability prevention activities in global programme to eliminate lymphatic filariasis.

**Summary:**

Disease specific Health Related Quality of Life instrument can be used to measure the longitudinal changes in quality of life of patients following the intervention. High responsiveness, clinical relevance to patients and its sensitiveness to detect small changes are the merits of disease specific instrument. Morbidity management and disability prevention activities under filariasis elimination programme aim at improving the quality of life of patients with irreversible manifestations. Therefore there is an urgent need to develop an instrument to assess the health related quality of life, specific for lymphatic filariasis by incorporating all the difficulties and problems caused to patients by the disease in the physical, mental and social domains of health.

## Background

Lymphatic Filariasis is an important public health problem in many tropical and sub-tropical countries. Globally, 1.3 billion people are at risk of infection and about 120 million people are affected in 83 countries [1]. This disease causes an estimated annual burden of 5.77 million DALYs [2] in its endemic countries. The Global Programme for the Elimination of Lymphatic Filariasis (GPELF) was established in early 2000 following the World Health Assembly Resolution 50.29 [3] in 1997. The GPELF has 2 components. First is to interrupt the transmission of infection through Mass Drug Administration (MDA) with Diethylcarbamizine (DEC) or co-administration with Albandazole. Second is to reduce LF-related disability in those, already affected by chronic manifestations of the disease through Morbidity Management and Disability Prevention Activities (MMDPA). MMDPA includes basic limb hygiene, which can prevent secondary infections causing the acute episodes (Adenolymphangitis) among lymphoedema patients, and surgical corrections for hydrocele cases. [4]. GPELF has launched MDA in 42 of the 83 endemic countries covering a population of 610 million in 2005. Rest of the 8 countries may not require MDA and 33 countries have not yet implemented the programme [1]. However, the morbidity management activity, the second component of the GPELF strategy is lagging [5]. Out of 83 endemic countries, only 27 have initiated the morbidity management programme [1]. To date, the primary focus of the GPELF has been on the chemotherapeutic interventions, that is MDA. The strategy for MDA has been clearly laid out and guidelines are well documented for implementation, monitoring and evaluation of the programme [6]. However the guidelines for monitoring and evaluation have not yet been formulated or standardized for the MMDPA. Hence there is an urgent need to develop the strategy and guidelines for monitoring and evaluating the MMDPA programme.

## Discussion

Although GPELF programme has gathered momentum, there are some issues to be resolved for achieving the desired results of the programme. One among them is the low priority given to morbidity management programme. The reason for this may be the limited availability of resources including trained personnel and surgical facilities for performing hydrocelectomies. The strategy for MDA has been clearly laid out and guidelines are available for monitoring and evaluating the MDA programme and are being followed by the implementing countries. The epidemiological indicators such as microfilaria (mf) prevalence and mf density levels are used to assess the impact of MDA programme [7]. Ultimately absence of transmission is verified using antigenemia in children. However, the guidelines for monitoring and evaluating the morbidity management programme have not yet been formulated or standardized and as of now no indicators/tools are available to assess the impact of morbidity management. The World Health Organization (WHO) has proposed to develop an instrument based on *International Classification of Functioning, Disability and Health *(ICF) and *WHO Disability Assessment Schedule (WHODAS) *[8] for assessing the impact of chronic disabling diseases like LF, leprosy, diabetes etc. The WHODAS was developed building on the experience of existing instruments developed to assess health and disability. It is a general health state assessment measure that can be used in epidemiological surveys, health systems research such as the evaluation of needs and outcomes, clinical assessment and as a potential descriptive system for Summary Measures of Population Health (SMPH). It gives a general disability score as well as different profiles on health domains like cognition, mobility, self-care, interpersonal relations and participation in community. However there are constraints in using WHODAS as an assessment tool for evaluating MMDPA for LF. It is a general health state assessment instrument and thereby ignores the entanglement of diseases and their signs or symptoms. Since the disabilities due to LF are dependent on its particular health state, this instrument may not be suitable to assess the impact of intervention programmes on LF. Further, MMDPA aims at improving the quality of life and therefore it is ideal to develop an instrument, which can measure the quality of life.

There is an increasing recognition that what matters most to patients is how well they are able to function in their day-to-day life [9] and the self-reported health status is receiving increasing attention in epidemiological and outcomes research [10]. Understanding the impact of chronic illness on functioning and well being in physical, mental and social dimensions of life have become essential and therefore efforts to incorporate quality of life in medical care outcome studies are increasing [11]. Health Related Quality of Life (HRQoL), a multidimensional construct referring to patients' perceptions of the impact of disease and treatment on their physical, psychological and social function and well being [12] is crucial in the evaluation of health care interventions [13]. LF has a wide clinical spectrum [14] and disability is more pronounced in the advanced stages of lymphoedema and Hydrocele. Since the advanced stages of lymphoedema are not reversible, MMDPA can lessen the disabilities due to secondary infections and there by improve the quality of life of the patient. Thus, an improvement in quality of life is considered to be important as a primary outcome in the determination of therapeutic benefit.

The concept of HRQoL is used as an important parameter for measuring outcome in modern medicine. This can be used as discriminative as well as an evaluative indicator. Therefore HRQoL can be considered as an indicator to assess the impact of MMDPA in GPELF. HRQoL can be measured using both generic and disease specific instruments [15]. Generic instrument assesses general aspect of HRQoL that are applicable to any disease or health conditions but lacks sensitivity to detect the changes after the intervention, whereas the disease specific instrument assess the particular concerns and conditions related to that particular disease or health state [16] and so is more sensitive to detect the changes between pre and post intervention period. Disease specific measures are most appropriate because of their higher responsiveness and their outcomes will be clinically relevant to the patient. In LF, disability is more pronounced in the advanced stages of lymphoedema and hydrocele. While disability due to hydrocele can be reverted through surgery, disability due to the advanced stages of lymph oedema is not reversible and hence disability due to these health states cannot be reduced. However it is possible to prevent secondary bacterial infections leading to ADL attacks through simple home-based approaches like leg hygiene and simple exercises. These measures will reduce the frequency of ADL attacks and thereby improve the quality of life of the patients. A generic instrument may not be able to capture all the items, which are potentially affecting their quality of life including the specific disabling signs and symptom of LF. Therefore it is suggested that LF specific instruments be utilized for assessing the impact. Since the disease specific HRQoL instruments are not available for LF, there is an urgent need to develop such a valid and reliable instrument to assess the impact of morbidity management programme.

## Summary

Guidelines for ELF programme implementation and monitoring are necessary for the programme managers at different levels. Such guidelines are already available for MDA, but warrant one for MMDPA. Monitoring the impact of intervention is an inbuilt component of the programme and is essential to assess the progress and endpoints. Though WHODAS has been recommended as a generic instrument, it can not be considered as more appropriate. HRQoL can be used as an indicator to assess the impact of MMDPA and LF specific HRQoL instrument (LF-QoL) is appropriate, as it can be developed to assess the health outcome in terms of quality of life. MMDPA aims at reducing the morbidity and improving the quality of life of chronic patients, which is irreversible. This is not only useful to assess the effect of home management to prevent secondary infection and progression of disease, but also to assess the impact of surgical corrections of hydrocele patients. LF-QoL can be developed following standard methods described elsewhere [12,16]. All the difficulties and problems caused to patients by the disease in the physical, mental and social domains can be incorporated in developing the questionnaire. As the morbidity pattern and response to morbidity varies with the type of manifestation, it is necessary to assess the HRQoL for different clinical manifestations of LF by LF-QoL instrument. Similar assessment can be done following the morbidity management interventions and the outcome can be compared prior to and after the intervention. This will indicate the level of improvement in the quality of life following the intervention because disease specific HRQoL measure is more sensitive to detect the changes between pre and post intervention period. MMDPA has not yet received much priority/attention by the programme managers as only 27 out of 83 endemic countries have initiated this programme. The primary focus of programme managers has been on to scaling up MDA to cover all at-risk populations. The countries implementing MDA to interrupt the transmission of filarial parasite should also establish programmes to implement the morbidity management to prevent LF related disability to achieve the overall objective of GPELF by the year 2020.

## Competing interests

The author(s) declare that they have no competing interests.

## Authors' contributions

AK drafted the original manuscript and currently working on the development of LF specific instrument to assess the quality of life; KK conceived the idea in developing potential alternative measure to assess the impact of morbidity management interventions against LF and reviewed the document; KTH assisted in organizing bibliographic data base and LKD contributed in developing the concept and reviewing the manuscript.
